# Chromosomal abnormalities and Y chromosome microdeletions in infertile men from Morocco

**DOI:** 10.1186/s12894-015-0089-3

**Published:** 2015-09-18

**Authors:** Yassine Naasse, Hicham Charoute, Brahim El Houate, Chadli Elbekkay, Lunda Razoki, Abderrahim Malki, Abdelhamid Barakat, Hassan Rouba

**Affiliations:** Laboratoire de Génétique Moléculaire Humaine, Département de la Recherche Scientifique, Institut Pasteur du Maroc, 1 Place Louis Pasteur, 20360 Casablanca, Morocco; Laboratoire de Cytogénétique, Département de la Recherche Scientifique, Institut Pasteur du Maroc, 1 Place Louis Pasteur, 20360 Casablanca, Morocco; Laboratoire de Physiopathologie et Génétique Moléculaire, Faculté des Sciences Ben M’Sik, Université Hassan II, Casablanca, Morocco

**Keywords:** Male infertility, Chromosomal abnormalities, Y microdeletions, Severe oligozoospermia, Azoospermia

## Abstract

**Background:**

Male infertility is responsible for 50 % of infertile couples. Thirty percent of male infertility is due to cytogenetic and genetic abnormalities. In Arab and North African populations, several studies have shown the association of these chromosomal abnormalities with male infertility. Our objective is to evaluate the frequency of chromosomal abnormalities and Y chromosome microdeletions in infertile men from Morocco.

**Methods:**

A total of 573 Moroccan infertile men (444 azoospermic and 129 oligozoospermic men) referred for cytogenetic analysis to the Department of Cytogenetics of the Pasteur Institute of Morocco, were screened for the presence of chromosomal abnormalities and Y chromosome microdeletions.

**Results:**

Chromosomal abnormalities accounted for approximately 10.5 % (60/573). Fifty six cases among them have sex chromosome abnormalities (93.34 %), including Klinefelter’s syndrome in 41 patients (68.34 %). Autosomal chromosome abnormalities (6.66 %) were observed in 4 patients. Chromosomal abnormalities were more prevalent in azoospermic men (13.06 %) than in oligospermic men (1.55 %). Y microdeletions were detected in 16 of 85 patients (AZFc: 14.12 %, AZFbc: 4.70 %), most of them where azoospermic men with no chromosomal abnormality.

**Conclusions:**

These results highlighted the need for efficient molecular genetic testing in male infertility diagnosis. In addition, a genetic screening should be performed in infertile men before starting assisted reproductive treatments.

## Background

Infertility is a public health problem; approximately 15 % of couples have difficulty or are unable to conceive [1]. Whereas, from 30 % to 50 % of infertility cases are due to a male factor [2]. There are many and diverse causes of male infertility, including accidental causes, congenital birth defects, functional impairments, environmental pollutants, or genetic factors. The latter represents between 10 % and 15 % of severe male infertility [3, 4].

Chromosomal anomalies are considered as one of the most important causes of male infertility [5–7]. Their incidence is higher in infertile men than the general population (about 10 times) [8]. Chromosomal abnormalities (structural or numerical) are often detected in azoospermia and severe oligospermia [9], at frequencies ranging from 10 % to 23.62 % and from 1.10 % to 13.33 %, respectively [8, 10–14].

Chromosomal abnormalities are not the only genetic causes of male infertility. Deletion of the Y chromosome region containing the azoospermia factor (AZF) is considered the most common genetic cause of male infertility [15]. Three regions on Y chromosome long arm (AZFa, AZFb and AZFc) are recurrently deleted in men with severe spermatogenic failure [16]. The frequency of these microdeletions in azoospermic and severe oligospermic men is between 1 % and 50 % [16–18].

This study aimed to assess the different types of Y chromosome abnormalities and microdeletions, and their frequency in a Moroccan group of infertile men. In addition, we conducted several comparisons between frequencies found in the current study and frequencies reported in other populations.

## Methods

### Patients

A group of 573 Moroccan infertile men were involved in this retrospective study. These patients with azoospermia or severe oligozoospermia were recruited at the Pasteur Institute of Morocco from 1998 to 2013. Patients with azoospermia or severe oligozoospermia resulting from endocrine or obstructive causes were excluded. The average age was 38.70 ± 7.00 years. The Karyotype was performed for all patients, while the screening for Y-chromosomal microdeletions was conducted in 85 patients for whom DNA samples were available.

### Ethics statement

We obtained written informed consent from each subject and the study protocol was approved by the local committee on research ethics of the Pasteur Institute of Morocco.

### Chromosomal analysis

Peripheral blood was collected in heparinized vacutainers. The cells were harvested and cultured in the laboratory of cytogenetics. Samples were incubated in an RPMI-1640 solution in the presence of phytohemagglutinin (PHA-E) for 72 h at 37 °C. 2 h before the end of the culture, colchicine was added. After centrifugation, the pellet recovered was treated with a hypotonic solution (0.075 M KCl). The samples were fixed by Carnoy fixative (acetic acid/methanol 1/3 acid). Fixed cell suspensions were spread on glass slides using a Pasteur pipette. These slides were immersed in the fixative Berger. The slides were immersed in the denaturing medium Earle. Then they undergone a Giemsa staining, and finally reading slides by G-banding technique using a microscope connected to a computer through a camera. At least 20 metaphases were counted for each sample.

### Y-microdeletions studies

The genomic DNA extraction was carried out using the Phenol Chloroform standard conventional protocol in the laboratory of Human Molecular Genetics at the Pasteur Institute of Morocco. The samples were lysed by incubation of erythrocytes in Tris-EDTA (TE 20/5) followed by centrifugation. Once the pellet was white, it has undergone lysis of leukocytes by incubation in a solution containing Tris-EDTA (TE 10/5), 20 % SDS and Proteinase K overnight at 56 °C. Phenol was added and the tube centrifuged. After recovery of the upper aqueous phase, the Chloroform-Isoamyl Alcohol (24/1) was added. After centrifugation, the upper aqueous phase was recovered. Absolute Ethanol was added for DNA precipitation. Once recovered and dried, the DNA was resuspended in Tris-EDTA (TE 10/1) 2 to 3 days and then diluted and stored at 4 °C before analysis.

Conventional microdeletions in the long arm of the Y chromosome (Yq) were detected by the polymerase chain reaction (PCR). 6 pairs of oligonucleotide primers were used to amplify the specific Y chromosome STS, which correspond to different loci of the azoospermia factors (AZF). Two sets of multiplex PCR reactions were performed for each patient. The primer pair that amplifies specific STS SRY gene was used as positive control.

The volume of the reaction mixture used in the multiplex PCR was 25 μl, containing 5 μl of DNA (25 ng/μl), 2.5 μl of MgCl_2_ buffer (10X), 2 μl of dNTP (2.5 mM) 0.9 μl of MgCl_2_ (25 mM), 0.2 μl of Taq DNA polymerase (5 units) and 0.8 μl of each primer (10 pM). On thermocycling, the Touch-Down PCR method was used to increase the specificity and reproducibility of the PCR by avoiding the presence of non-specific bands. The program used an initial denaturation of 5 min at 94 °C, followed by 5 cycles of 40 s at 94 °C, 45 s at 60 °C and 50 s at 72 °C, followed by 30 cycles of 35 s at 94 °C, 35 s at 58 °C and 40 s at 72 °C, and finally a final elongation of 7 min at 72 °C. Electrophoresis was performed on 3 % agarose gels containing Ethidium Bromide (EtBr) was performed for the control of the PCR products, using a size marker 100 bp. The gel was visualized under ultraviolet light.

## Results

This study was performed on a sample of 573 infertile men, which were separated into two groups: men with non-obstructive azoospermia (*n =* 444; 77.48 %), and men with severe oligospermia (*n =* 129; 22.52 %) (Table 1). The average age is 38.70 ± 7.00 years. Cytogenetic analysis showed that among the 573 patients included in the study, 513 men (89.5 %) had a normal karyotype (46; XY), and 60 men (10.5 %) had various chromosomal abnormalities. Frequencies of these chromosomal abnormalities in patients with non-obstructive azoospermia and severe oligozoospermia, are 10.12 % (*n =* 58) and 0.35 % (*n =* 2), respectively. Among the 60 patients with chromosomal abnormalities, four (0.7 %) had an abnormality of autosomal chromosomes: Inversion 46; XY, inv 9 (p11; p13), and 56 men (9.8 %) had sex chromosome abnormalities: two mosaic forms 46; XY/47; XXY, one patient with 47; XYY, three patients with 45; X (15 %)/46; XY (85 %), three patients with 46; X, del Yq11, six patients with 46; XX, and the most frequent is Klinefelter syndrome (KFS) 47; XXY, it was found in 41 patients (39 azoospermic men, and two men with severe oligozoospermia) (Table 2).

**Table 1:** Distribution of different types of infertility

Type of infertility	% (No. of men/Total No.)
Non obstructive Azoospermia	77.48 (444/573)
Oligozoospermia Severe	22.52 (129/573)

**Table 2:** Type and frequency of chromosomal anomalies in 573 Moroccan infertile men

	Non-obstructive Azoospermia	Severe Oligospermia	Overall those with counts < 5 million/ml
Sex Chromosome			
Aberrations			
47 ; XXY	8.79 % (39/444)	1.55 % (2/129)	7.16 % (41/573)
Mosaic 46 ;XY/47 ;XXY	0.46 % (2/444)	0 % (0/129)	0.35 % (2/573)
47 ;XYY	0.23 % (1/444)	0 % (0/129)	0.18 % (1/573)
45 ;X(15 %)/46 ;XY(85 %)	0.68 % (3/444)	0 % (0/129)	0.53 % (3/573)
46 ;X, del Y q11	0.68 % (3/444)	0 % (0/129)	0.53 % (3/573)
XX males (46 ;XX)	1.36 % (6/444)	0 % (0/129)	1.05 % (6/573)
Subtotal	12.2 % (54/444)	1.55 % (2/129)	9.8 % (56/573)
Autosomal Chromosome			
Abnormalities			
Inversion	0.9 % (4/444)	0 % (0/129)	0.7 % (4/573)
46 ;XY, inv 9 (p11 ;p13)			
Total	13.06 % (58/444)	1.55 % (2/129)	10.47 % (60/573)

Molecular analysis was performed to detect Y chromosome microdeletions in 85 patients. Among these patients, twelve had a deletion of AZFc (14.12 %), four cases had a deletion of AZFbc, and 69 cases had an intact Y chromosome (81.18 %). No patient with a deletion of AZFa, AZFb or AZFabc was observed (Table 3). The occurrence of AZFc locus deletion is identical among azoospermic and oligospermic (50 %), whereas the AZFbc deletion was found in 75 % of azoospermic and 25 % of oligozoospermic patients (Table 3).

**Table 3:** Type and frequency of Y-microdeletions in 85 infetiles men evaluated

	Non-obstructive azoospermia	Severe oligospermia	Overall those with counts < 5 million/ml
Absence of Y-microdeletions	76.81 % (53/69)	23.19 % (16/69)	81.18 % (69/85)
AZFc microdeletion	50 % (6/12)	50 % (6/12)	14.12 % (12/85)
AZFbc microdeletion	75 % (3/4)	25 % (1/4)	4.70 % (4/85)

All cases with AZFc or AZFbc locus deletion had a normal karyotype (46; XY). For patients with no deletion (*n =* 69), only one case had the Klinefelter syndrome (47; XXY). The remaining patients had a normal karyotype (Table 4).

**Table 4:** Incidence of Y-microdeletions in men with normal karyotype and men with Klinefelter Syndrome

	46 ; XY	47 ; XXY
Absence of Y microdeletions	98.55 % (68/69)	1.45 % (1/69)
AZFc deletion	100 % (12/12)	0 % (0/12)
AZFbc deletion	100 % (4/4)	0 % (0/4)

## Discussion

Numerical and structural chromosome abnormalities played a principal role in male infertility. The prevalence of these abnormalities remained poorly studied and little discussed in the literature, including their prevalence in the Moroccan population.

Studies performed in different populations showed that the incidence of chromosomal abnormalities in infertile men varies between 2.2 % and 19.6 % [19]. In the current study, the frequency of chromosomal abnormalities in infertile men (non-obstructive azoospermic and severe oligozoospermic men) was 10.5 %. This frequency is comparable with those reported in Table 5. It is well known that the sperm count is inversely related to the presence of chromosomal abnormalities [20]. The proportion of chromosomal abnormalities in azoospermic men (13.06 %) was significantly higher than in severe oligospermic men (1.55 %), these frequencies were consistent with those found in other studies [20].

**Table 5:** Comparison of chromosomal anomalies between this study and other similar studies

Authors	Regions	No. of cases	Frequencies %	Prevalence of chromosomal aberration % (No. of men/total No.)	
				Non-obstructive Azoospermia	Severe Oligospermia Counts < 5 million/ml
Tuerlings et al., 1998 [45]	Netherlands	968	3.51	6.45 % (4/62)	3.47 % (30/865)
Nagvenkar et al., 2005 [11]	India	88	10.22	14.29 % (6/42)	6.52 % (3/46)
Mohammed et al., 2007 [33]	Kuwait	289	7.95	19.44 % (21/108)	1.10 % (2/181)
Ng et al., 2009 [12]	Hong Kong	295	5.08	21.10 % (5/71)	4.46 % (10/224)
Kosar et al., 2010 [5]	South of Turkey	115	4.34	5.43 % (5/92)	0 % (0/23)
Alkhalaf et al., 2010 [46]	Kuwait	142	18.30	data not available	data not available
Mafra et al., 2011 [34]	Brazil	143	6.29	11.62 % (5/43)	4 % (4/100)
Zhang et al., 2012 [32]	Northeast China	135	14.07	17.28 % (14/81)	9.26 % (5/54)
Cavkaytar et al., 2012 [47]	Turkey	332	7.23	11.22 % (22/196)	1.47 % (2/136)
Amouri et al., 2014 [30]	Tunisia	476	10.92	14.10 % (46/328)	4.05 % (6/148)
Our study	Morocco	573	10.47	13.06 % (58/444)	1.55 % (2/129)

No.: number

According to the study conducted by Ferlin et al., Klinefelter’s syndrome frequency in infertile men reached respectively 10 % and 5 % in azoospermic and severe oligospermic men [4]. In our patients, we found that the Klinefelter syndrome 47; XXY was the most common in azoospermic men (9.25 %) than in oligospermic severe men (1.55 %). In consistence with other studies [21, 22], the mosaic forms of Klinefelter syndrome was found only in two azoospermic patients. Most men with KS are azoospermic and their ability to conceive remained too low [23]. However, Klinefelter Syndrome patients may have their own children genetically using the technique of testicular sperm extraction (TESE) or micro-TESE followed by ICSI [24]. However, their offspring have an increased risk for chromosomal abnormalities [25].

Structural rearrangements of the Y chromosome, including deletions, ring chromosome and isochromosomes may lead to different phenotypes. In this study, we detected three patients with deletion of the Y chromosome long arm, which are all azoospermic men. In fact, the long arm of the Y chromosome plays a crucial role in the process of spermatogenesis. Partial or total loss of the arm means the loss of genes controlling spermatogenesis, leading to a defect in sperm production [26, 27].

Disorders of sex development (DSD) regroup the clinical situation where the development of the chromosomal, gonadal or anatomical sex is atypical [28]. Three form of the DSD sex caused by chromosome aneuploidy were observed in our sample: The XX male, 47; XYY and 45; X/46; XY.

The “XX Male Syndrome” is one of the sex chromosome abnormalities. Men with this syndrome can have phenotype similar to those with Klinefelter’s syndrome with normal male external genitalia, but they are sterile [29]. Studies have shown that the frequency of XX males among azoospermic varies between 0.6 % [30] and 3.7 % [21, 22]. In our study, the frequency of this chromosomal abnormality was 1.36 %.

Three patient have the mosaic 45; X (15 %)/46; XY (85 %), and one patient have 47; XYY, all of them are azoospermic. The mosaic form 45; X (15 %)/46; XY (85 %) are described in many studies and the phenotype can range from female phenotype and ambiguous to male phenotype with infertility [30]. The 47; XYY syndrome is associated with behavior difficulties and in some case with infertility. Most males born with these chromosome patterns will go through life without being karyotyped [31]. Until now, no association was found between increased frequency of infertility and this syndrome.

Autosomal abnormalities are less frequent than sex chromosome abnormalities: 0.7 % versus 9.8 %. On the other hand, autosomal chromosome abnormalities are detected only in azoospermic men (0.9 %). These results are not similar with those reported in other studies [12, 30, 32]; Amouri et al. reported that gonosomal abnormalities are more common in azoospermic men, while autosomal abnormalities are more common in severe oligospermic men [23].

We identified 4 cases of autosomal chromosome abnormalities (0.7 %), all of them have the inversion 46; XY, inv 9 (p11; p13). This inversion even balanced may be a factor of structural chromosome aberrations during meiosis, and by the way a cause of the disturbances of spermatogenesis.

After the Klinfeleter syndrome, Y chromosome micodeletion are the second most frequent genetic cause of infertility. The frequencies of Y chromosome microdeletions differ from one study to another, and this may be due to several factors such as the choice of inclusion criteria, the STS markers used, and/or ethnicity of the study population [33, 34]. In our study, sixteen patients among the 85 analyzed infertile men showed the presence of Y microdeletions (18.83 %). This frequency is in accordance to that found by Ghorbel et al. (17.1 %) [21], Fayez et al. (20.4 %) [22], Imken et al. (3.15 %) [35] and El Oualid et al. (3.83 %) [36], although our cohort of patients showed a higher frequency, and this may be due to the low number of patients ascertained as well as the high number of patients with Y microdeletions, but higher than that found in the Chinese population (6.4 %; 8.5 %; 9.1 %) [12, 37, 38] or other Arab populations (Kuwait (2.6 %) [33], Tunisia (2.7 % and 6.85 %) [39, 40], Saudi Arabia (3.2 %) [41] and Egypt (4 %) [42]. However, the frequency found in this study was included in the range of frequencies of wide world (1–55 %) [17, 18]. These microdeletions are more common in azoospermic (*n =* 9) than severe oligospermic (*n =* 7), and this proportion was found in many reports [33, 35, 37–40, 42].

In this report, we identified only deletions of the AZFc and AZFbc region, while no case with deletion of AZFa and/or AZFb was identified. This result is similar to that described in Ng et al. [12] and Imken et al. [35] reports, but different to the results found in other reports [17, 34, 42, 43]. Several studies have shown that AZFc deletion was the most common [22, 39, 40], and these results are in accordance with ours (14.12 %). However, other studies showed conflicting results [33, 35, 42, 44].

In this study, we showed that sixteen cases with microdeletions (AZFc and AZFbc) have no chromosomal abnormalities (normal karyotype 46; XY) (Table 4). While Ng et al. showed that all patients with AZFc deletion had a normal karyotype 46; XY, while all patients with AZFbc deletion had chromosomal abnormalities [12].

## Conclusion

The prevalence of chromosomal abnormalities and Y chromosome microdeletions are comparable to that found in different parts of the world (North Africa, Asia and Europe). Indeed, the analysis of these genetic factors (karyotype, Y microdeletions, etc. …) is highly recommended, to identify the causes of infertility and to choose the appropriate assisted reproduction technique, and also to reduce the risk of transmission of these genetic defects to the future generations.

## Availability of data and materials

Not applicable.

## Abbreviations

AZF: Azoospermia Factor
dNTP: Triphosphate Deoxyribonucleotides
DSD: Disorders of Sex Development
EDTA: Ethylenediaminetetraacetic acid
BET: Ethidium Bromide
ICSI: Intracytoplasmic Sperm Injection
KCl: Potassium Chloride
KFS: Klinefelter Syndrome
PCR: Polymerase Chain Reaction
PHA-E: Phytohemagglutinin- Erythrocytes
RPMI: Roswell Park Memorial Institute medium
SDS: Sodium Dodecyl Sulfate
SRY: Sex-determining Region of Y chromosome
STS: Sequence-tagged site
TE: Tris-EDTA
TESE: Testicular Sperm Extraction
